# Evaluation of Anticancer Activity of *Satureja montana* Supercritical and Spray-Dried Extracts on Ehrlich’s Ascites Carcinoma Bearing Mice

**DOI:** 10.3390/plants9111532

**Published:** 2020-11-10

**Authors:** Jelena Vladić, Tatjana Ćebović, Senka Vidović, Stela Jokić

**Affiliations:** 1 Department of Biotechnology and Pharmaceutical Engineering, Faculty of Technology, University of Novi Sad, Bulevar cara Lazara 1, 21000 Novi Sad, Serbia or vladicj@uns.ac.rs (J.V.); senka.vidovic@uns.ac.rs (S.V.); 2 Department of Biochemistry, Faculty of Medicine, University of Novi Sad, Hajduk Veljkova 3, 21000 Novi Sad, Serbia; 3 Faculty of Food Technology Osijek, Josip Juraj Strossmayer University of Osijek, Franje Kuhača 20, 31000 Osijek, Croatia

**Keywords:** *Satureja montana*, winter savory, cytotoxic activity, Ehrlich ascites tumor, supercritical carbon dioxide, spray drying, carvacrol

## Abstract

*Satureja montana* herbal species belongs to aromatic medicinal plants with a significant place in traditional medicine. However, products produced with conventional procedures do not meet the requirements of the modern market which include environmentally-safe processes that provide quality, safe, and standardized products. In this study, the antiproliferative activity of *S. montana* extracts obtained by supercritical carbon dioxide and solid–liquid extraction followed by spray drying was investigated using the in vivo model of Ehrlich ascites carcinoma (EAC) in mice. The impact of two concentrations of extracts on the growth of tumor and the redox status of malignant cells was monitored. It was determined that the extracts induced oxidative stress in the malignant cells which was confirmed by the changes in activity of biochemical indicators of oxidative stress. The posttreatment was not an efficient approach, while the extracts applied as pretreatment and treatment resulted in an increase in the xanthine oxidase (XOD) activity, a decrease in catalase (CAT) activity, and an increase in the intensity of lipid peroxidation (LPx). Furthermore, a decrease in the values of reduced glutathione (GSH) and an increase in glutathione reductase (GR) and glutathione peroxidase (GSHPx) in EAC cells were recorded.

## 1. Introduction

Winter savory (*Satureja montana* L.) represents an aromatic plant from the Lamiaceae family. The benefits of *S. montana* have long been recognized in traditional medicine which has established the plant as a widespread folk remedy. Furthermore, winter savory is used as a spice. *S. montana* is rich in essential oil with oxygen-containing phenolic monoterpenes carvacrol and thymol as its most dominant components [1]. In addition to lipophilic fraction, winter savory possesses significant bioactive components in its content which are hydrophilic in character, such as phenolic acids [2].

It was determined that the most dominant components of winter savory, isomers carvacrol and thymol, exhibit numerous beneficial biological activities, such as antioxidative, antimicrobial, diuretic, anti-HIV-1, antidiarrheal, and antiproliferative activities [3,4,5,6,7,8]. Very often, the biological activity of herbal products is attributed to its most dominant components. However, the effect of the herbal products due to the synergy of all the components present in the plant can be more significant than the activity of an individual component [9]. Moreover, the body’s response to the extract is not the same as the response to individual components.

The induction of apoptosis of cancer cells without significant side effects is a characteristic of a good chemoprotective agent [10]. Therefore, herbal extracts represent excellent candidates for research in this area as potential non-toxic agents. Herbal polyphenols demonstrated to be significant potential chemopreventive and chemotherapeutic agents considering that they can lead to the suppression of malignant proliferation with different mechanisms. Additionally, they can have an effect on the reduction of consequences of oxidative stress. For certain herbal species that belong to the *Satureja* genus, it was determined that they exhibit antiproliferative activity. Examples of those species are: *Satureja thymbra* and *Satureja parnassica* [11], *Satureja bakhtiarica* [12], and *Satureja khuzistanica* [13]. Furthermore, the antitumor activity of carvacrol was demonstrated in numerous studies [14,15,16,17,18]. Additionally, it was found that *S. montana* extracts exhibit antiproliferative activity on cancer cells [19,20]. With regard to investigating the activity of *S. montana* extracts on an in vivo model however, no study has yet been conducted.

Furthermore, the demand for natural products which is, along with market competition, constantly on the rise, dictates the demand for introducing new, modern, efficient, and green procedures which are to provide a stable, quality, and safe product. Modern production processes have to meet the following requirements of the modern market: production without the use of toxic solvents, extended stability of products, optimized chemical composition with efficient use of natural resources, reduced use of energy, higher process safety, and reduction of waste. The attainment of winter savory extracts was investigated by applying different modern green techniques and alternative solvents including supercritical and subcritical fluids [21,22,23,24,25], eutectic solvents [26], microwave-assisted extraction, and hydrodistillation [27,28].

For the aforementioned reasons, this study investigated the antiproliferative activities of *S. montana* extracts obtained by applying environmentally-friendly techniques and using the model of induced Ehrlich ascites carcinoma (EAC) in mice. Considering that winter savory possesses significant bioactive components that are polar and non-polar in character, lipophilic extracts obtained by supercritical carbon-dioxide extraction (Sc-CO_2_) and extracts with a more polar character obtained by solid–liquid extraction with ethanol followed by the spray drying process were investigated. To determine the potential of extracts, parameters of tumor growth and the antioxidant status of malignant cells were monitored. In addition, the impact of extracts before, at the time of, and after the implantation of EAC cells was monitored.

## 2. Results

### 2.1. Impact of S. montana Extracts on the Volume of Ascites, Viability, and Number of Cells

**Figure 1:**
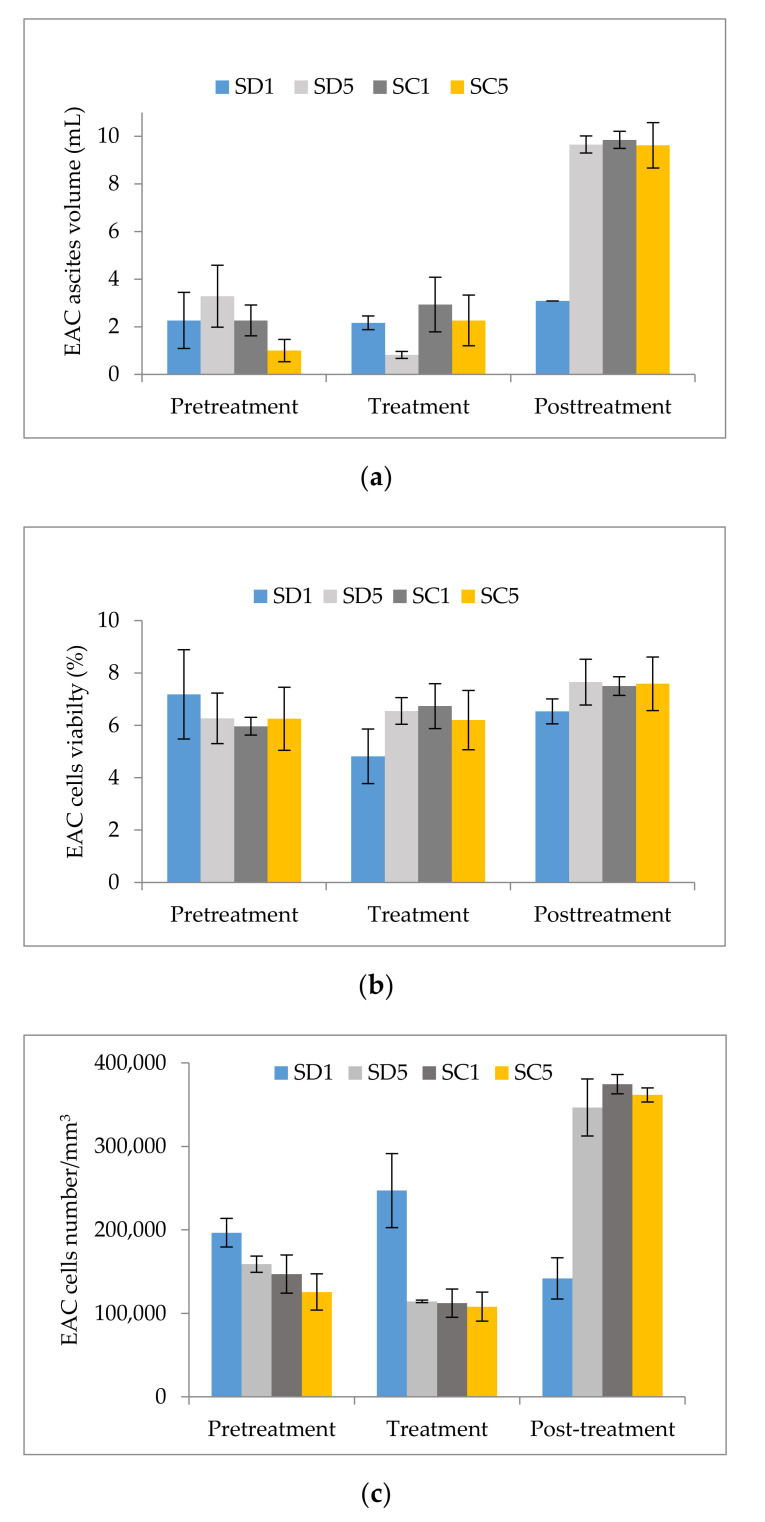
Impact of *S. montana* extracts on (**a**) Ehrlich ascites carcinoma (EAC) ascites volume, (**b**) EAC cells viability, and (**c**) EAC cells number (SD—spray-dried extract; SC—extract obtained by Sc-CO_2_).

In order to determine the activity of *S. montana* extracts on the parameters of tumor growth, the changes in cell parameters were monitored—ascites volume, number of tumor cells, and tumor cells variability (Figure 1; Table A1, Table A2 and Table A3). The impact of spray-dried (SD) extract and extract obtained by Sc-CO_2_ (SC extract) was investigated in two concentrations (1% (SD1 and SC1) and 5% (SD5 and SC5)) and different impacts of the extracts on the volume of ascites were recorded (Figure 1a; Table A1). After the application of extracts in the pretreatment (both extracts and both concentrations), there was a significant decrease in the volume of ascites compared to the EAC control group. Moreover, the most significant decrease was recorded in the group that was pretreated with SC5. During treatment, there was also a significant decrease in the volume of the ascites when all extracts were applied. In addition, the most efficient decrease among the treated groups was recorded in a group in which SD5 was applied. In the posttreatment, a reduced volume was only recorded in a group in which SD1 was applied, while in all other groups an increase in the volume of ascites compared to the EAC group was recorded.

The groups which were pretreated and post-treated with extracts, a significant change in variability was not recorded (Figure 1b; Table A2). In the groups of animals that were treated, a statistically significant decrease of cell variability was recorded only after the treatment with the SD1 extract, while in all other treated groups of animals, there was a statistically insignificant decrease in variability.

The impact on the number of cells compared to the EAC control group was statistically significant only in groups of animals that were pretreated with SD and an increase in the number of cells was determined. In the treatment with SD1, the same effect was recorded—a significant increase in the number of cells, while the administration of SC extracts led to a mild reduction (statistically insignificant) in the number of malignant cells. In the post-treated groups, there was an increase recorded after the application of all extracts (Figure 1c; Table A3).

### 2.2. Impact of S. montana Extracts on the Antioxidant Status Malign Cells of EAC

The changes in the antioxidative status of EAC cells were monitored by measuring the activity of antioxidative enzymes (XOD, CAT, Px, GSHPx, and GR) and the quantity of GSH and the intensity of LPx in EAC cells. The obtained results were compared with the control group, that is, with the results of measurements of the same parameters in EAC cells (Table 1).

In the control group, the XOD activity in EAC cells was low, and by applying the SC extracts in pretreatments, there was a significant increase in the XOD activity in malignant cells, without a difference in their impact on this parameter (SC1 and SC5). On the other hand, the application of SD extracts did not cause any significant changes in the intensity of XOD. In the groups treated with extracts, significant changes in the activity of enzymes were determined where SC extracts intensified the XOD activity in EAC cells, as well as in the treatment with SD5. Furthermore, the treatment with a lower concentration of SD significantly decreased the activity of XOD. The application of extracts in post-treatments caused significant changes in all groups (except in the posttreatment with SD1)—a decrease in XOD activity compared to the EAC group, while the post-treatment with SD1 caused an increase in the XOD activity.

Compared to the activity of CAT in the EAC control group, the application of extracts resulted in a different impact on the activity of this enzyme. In the groups pretreated with extracts, the activity of CAT in EAC cells was significantly increased with the application of SD1, while it was significantly decreased in other pretreated groups. Moreover, the highest decrease was achieved when SC5 was applied in the pretreatment. The treatment with extracts in all animal groups caused significant changes in the CAT activity. In the pretreatment, the application of SD1 increased the CAT activity, while all other extracts caused a decrease in the activity of CAT of malignant cells. The application of extracts in post-treatments in all groups caused significant changes compared to the EAC control group in terms of decreasing the CAT activity with posttreatment with SD1, and an increase in the remaining three groups of post-treated mice.

The activity of Px in malignant cells compared to the control EAC group increased after the application of SC extracts and SD5 in pretreatments. The treatment with SD1 resulted in a decrease in the activity of Px, and an increase was determined in the treatments with SD5, SC1, and SC5. In post-treated groups, the SD1 extract caused changes in the intensity through the increase in the Px activity, while SD5 and SC decreased the activity of Px in EAC cells.

The activity of the GR enzyme in tumor cells was significantly different after the application of extracts. SC extracts and SD5 in the pretreated groups caused an increase in the activity of GR. Moreover, the highest increase was recorded in the group of mice pretreated with SC5. By starting therapy at the moment of tumor implantation, there were significant changes in the activity of enzymes in EAC cells. The treatment with SC and SD5 resulted in an increase, while treating mice with SD1 resulted in a decrease in the activity in the GR in EAC cells. After the application of SD1 in post-treated groups of animals, an increase was recorded, while the post-treatment with other extracts resulted in a decrease in the activity of GR.

The GSPHx activity in EAC cells when extracts SD5 and SC were applied prior to the implantation of tumor, increased compared to the control group. The impact of extracts that were applied as a treatment was also significant. It was determined that there was an increase in this activity in all treated groups (SC1, SC5, and SD5), except for the group treated with SD1 where the GSHPx activity was significantly decreased. The post-treatment of animals with SC1, SC5, and SD5 extracts caused a decrease in the GSPHx activity, and an increase with the application of SD1.

Compared to the EAC control group, the amount of GSH was reduced in all groups that were pretreated. Moreover, a significant reduction in GSH was recorded in groups in which SC extracts were applied. Furthermore, animal treatment with extracts also resulted in a decrease in the amount of GSH, and SC extracts in the treatment also led to a significant reduction in the amount of GSH compared to SD extracts. In post-treatments, a reduction was recorded only in the case of SD1, while with SC extracts and SD5, an increase in the amount of GSG was determined.

The intensity of the lipid peroxidation in the group with EAC was low. In the application of extracts in the pretreatment, only SC extracts led to a significant increase in the intensity of the lipid peroxidation. The treatment with extracts did not have a statistically significant impact on the changes in intensity of LPx. By post-treating mice with extracts, the changes in the intensity of LPx were significant, so the post-treatment with SD1 resulted in an increase, while others caused a decrease in the intensity of LPx.

## 3. Discussion

The study investigated the antiproliferative activity of *S. montana* extracts obtained by different techniques of extraction. Modern extraction techniques were selected which, once the process is established, can easily be scaled-up to an industrial level and provide high-quality extracts while protecting the environment. The Sc-CO_2_ extraction represents the method of choice for the isolation of lipophilic components [29]. The spray drying process has proved to be the superior and efficient method because it is multiple times cheaper compared to freeze drying and provides extracts of extended stability with the possibility of concealing unpleasant odors and tastes. In addition, the smaller volume of extracts compared to liquid ones further contributes to lower expenses of storage and transport. Furthermore, dry extracts are characterized by instantaneous solubility and they can easily be incorporated in other products like pills and tablets and instant teas [30,31].

In the previously published studies by our research group, the procedures for obtaining extracts of winter savory with optimal physical–chemical characteristics were established [32,33]. Sc-CO_2_ (350 bar and 50 °C) was applied to obtain extracts that are lipophilic in character and contained the highest content of carvacrol [32]. In addition, the mixture water/ethanol was used for the extraction of predominantly polar components and spray drying was applied further to obtain powder [33]. The goal was to evaluate the antitumor activity of extracts of winter savory of polar and non-polar character to identify the potential mechanism of activity of extracts. Furthermore, to determine whether carvacrol as the most dominant component in winter savory is responsible for the manifestation of the activity, essential oil was isolated from powder and its chemical profile was determined [33].

Considering that the Sc-CO_2_ is a solvent that extracts only non-polar components while the mixture water/ethanol shows an affinity toward more polar components, the content of carvacrol in supercritical extract was expectedly multiple times higher compared to the powder (SC 60.82 g/100 g; SD 902.52 mg/100 g), according to the GC-FID (gas chromatography with flame-ionization detection) analysis [32,33]. Furthermore, the presence of other less represented compounds was recorded in essential oil isolated from the *S. montana* powder and supercritical extract, such as *p*-cymene, trans-caryophyllene, caryophyllene oxide, linalool, and terpinen 4-ol (Table 2). The presence of lipophilic components in powder was not dominant and a higher presence of polyphenols was expected. Polyphenolic components that were identified in the extracts of winter savory are caffeic, syringic, rosmarinic, gallic, ferulic, cinnamic, syringic, gentisic, ferulic, and vanillic acids, as well as luteolin, rutin, epicatechin, catechin, and quercetin [34,35].

The antioxidative activity of extracts was investigated by using the DPPH (2,2-diphenyl-1-picryl-hydrazyl-hydrate) assay. By comparing the manifested activities, expressed as the IC_50_ value; that is, the concentration with which 50% of free radicals is inhibited, the dry extract showed a stronger antioxidative activity (SC 17.40 µg/mL; SD 5.24 µg/mL) [33,36]. The extract with the more dominant percentage of carvacrol did not exhibit a stronger antioxidative activity. An explanation for this can be that carvacrol is not the main carrier of antioxidative activities in the extract. Additionally, Serrano et al. [37] reported a stronger antioxidant activity of ethanolic and aqueous extracts compared to essential oil. A reason they state the presence of a higher content of phenolic acids, such as rosmarinic and others, is that, because of their hydroxyl group, they have a strong capability of capturing free radicals. Further, it is suggested that the presence of sesquiterpenes, which generally have a lower antioxidative capacity, contributes to the lower antioxidative activity of oils [37].

In the studies which in their focus have the investigation of antitumor potential of *S. montana* extracts, the precise mechanism responsible for the antitumor activity has not been determined. The antiproliferative activity of different extracts of *S. montana* was investigated on HeLa (human cervix adenocarcinoma) [19,20], HT-29 (human colon adenocarcinoma) [20], and MCF-7 (human breast adenocarcinoma) [20], K562 (human chronic myelogenous leukemia cells [19], and MDA-MB-453 (breast cancer cells) [19]. A strong antioxidative capacity of *S. montana* extracts was confirmed along with their antiproliferative properties that were different depending on the type of extract. The authors made an assumption that *S. montana* as a strong antioxidant can impact the redox condition of cells which leads to decreased cell proliferation. A low level of free oxygen species is necessary to promote cell proliferation and redox changes have a significant role in the signal traductional pathway, which is important for the regulation of growth of cells [20]. Additionally, good selectivity with respect to activity was determined, especially towards K562 cells in comparison to normal MRC-5 human fibroblasts [19]. It was suggested that cytotoxicity is not the only thing responsible for the manifestation of the antitumor activity, but also the possible prenylation of proteins including Ras [17], as well as the antioxidant nature of carvacrol [38].

Furthermore, carvacrol is a confirmed antitumor agent. It was demonstrated that supplementation with carvacrol exhibits the antitumor effect on liver cancer induced with diethylnitrosamine in Wistar albino rats, most likely protecting the antioxidant defense system and preventing lipid peroxidation and damage of liver cells [18]. Recent studies demonstrate that the antiproliferative effect of carvacrol on metastatic cells of breast cancer (MDA-MB 231) is based on the activation of classic responses that belong to the mitochondrial pathway of apoptosis [39]. In addition, it was determined that carvacrol can induce apoptosis in cell lines of the hepatocellular carcinoma and the results suggest that the induction of apoptosis can be performed through direct activation of the mitochondrial pathway, and mitogen-activated protein kinase can have a significant role the antitumor effect of carvacrol [40].

Ascites fluid is highly important for the development of tumor considering that it represents a source of food for its cells; therefore, the increase in volume of ascites is a significant indicator of proliferation of tumor cells [41]. It was determined that the application of extracts leads to a decrease in volume of ascites in groups of mice, but not in the number of cells. Therefore, it can be assumed that in the decreased volume of ascites there is a concentrated higher number of cells and that extracts do not exhibit an impact in that respect; that is, they do not lead to the death of cells nor the inhibition of cell growth. Hence, they do not exhibit cytotoxic nor cytostatic activity with regard to malignant cells. The potential oncostatic effect (statistically insignificant) was exhibited only by SC (SC1 and SC5) and SD5 extracts applied as treatment.

It can be noted that SC extracts applied as pretreatment and treatment exhibited a more dominant effect on SD. The content of carvacrol in SC extracts is significantly higher compared to SD; hence, that could be the reason for SC extracts’ higher impact on the antioxidant status of EAC cells. Furthermore, when they were applied as treatment, SC extracts resulted in the reduction in the volume of ascites (statistically insignificant) and the decrease in malignant cells. On the other hand, SC extracts applied as pretreatment did not have an impact on the decrease of number of malignant cells so they cannot be considered as cytotoxic or cytostatic agents.

In the groups of animals that were post-treated with SC1, SC5, and SD5, it is possible that there was an occurrence of a higher level of development of the implanted tumor before the application of extract. Based on the results of the activity of enzymes (XOD, CAT, Px, GSHPx, and GR), the amount of GSH, and intensity of LPx, as well as the results of the volume of ascites and the number of cells in the mentioned groups of animals, it can be concluded that post-treatment of animals is not an adequate therapeutic approach for EAC. Therefore, the time of the application of extracts is of high importance.

During the development of tumor, the cells become resistant due to constant exposure to oxidative stress and they develop strong mechanisms of antioxidative protection [42,43]. The intensity of LPx, as well as the XOD and CAT activities in EAC cells was low. However, after the application of certain extracts, it was observed that there was an increase in the XOD activity, decrease in the CAT activity, and an increase in the intensity of LPx. Moreover, it was recorded that there was a decrease in the values of GSH in EAC cells in all groups of examined animals (except for groups post-treated with SC1, SC5, and SD5), as well as an increase in GR and GSHPx in malignant cells after the application of *S. montana* extract. These enzymes represent markers of oxidative stress and their increased activity suggests that due to the application of certain extracts, malignant cells were exposed to oxidative stress. As a result, it was determined that extracts of *S. montana* induce the production of reactive oxygen species in malignant EAC cells. However, the exact mechanism of activity was not clarified and further research is necessary.

Additionally, Table 3 contains values of biochemical parameters of oxidative stress measured in the control group that was treated with distillated water and in groups that were treated only with SC1 and SD1 extracts [36]. By comparing these values with the parameters of oxidative stress determined in this study, it can be concluded that extracts exhibit selectivity towards tumor cells by inducing them into oxidative stress, while not manifesting any side-effects on the healthy cells.

## 4. Materials and Methods

### 4.1. Plant Material

Aerial parts of winter savory (*Satureja montana*) were collected at the Institute of Field and Vegetable Crops, Backi Petrovac, Republic of Serbia, in July 2012. The collected plant material was naturally dried and then stored in paper bags at room temperature.

### 4.2. Preparation of Extracts

The detailed procedure of obtaining *S. montana* extract (SC) by Sc-CO_2_ extraction was described by Vladić et al. [32]. Briefly, the extraction of *S. montana* herbal material was conducted using Sc-CO_2_ at a pressure of 350 bar and a temperature of 50 °C. The extraction time was 4.5 h. The separator conditions were 15 bar and 23 °C [32].

The detailed procedure of obtaining *S. montana* extracts via spray drying is described by Vidović et al. [33]. Spray-dried extract (SD) is produced by drying liquid extracts via spray drying (inlet temperature 120 °C, outlet temperature 80 °C). *S. montana* liquid extracts were obtained using a 50% ethanol mixture as an extraction solvent. The extraction was carried out for five days at room temperature in a dark place. Maltodextrin (DE16) in the percentage of 10% (calculated on extract dry weight) was used as a carrier material [33].

### 4.3. Chemical Analysis

Gas chromatography–mass spectrometry (GC/MS) and gas chromatography with flame-ionization detection (GC/FID) analyses were performed according to the procedures described in previous studies [32,33]. GC analysis was performed on an Agilent GC6890N system coupled with a mass spectrometer model Agilent MS 5795. An HP-5MS column (30 m length, 0.25 mm inner diameter, and 0.25 μm film thickness) was used. Essential oil was isolated from SD powder using the hydrodistillation procedure according to the European Pharmacopoeia (Clevenger-type apparatus) and its chemical profile was determined. The injected volume of the sample solution (SD in methylene chloride and SC in methanol) was 5 μL with a split ratio of 30:1. Aromatic compounds were identified using the NIST 05 and the Wiley 7n mass database. The GC/MS operating conditions were as follows: injector temperature 250 °C, temperature program 60–150 °C (4 °C/min), carrier gas He with flow rate 2 mL/min. Quantification of carvacrol was performed with an FID detector and the calibration curve for carvacrol. The GC/FID operating conditions were: injector temperature 250 °C, temperature program 60–150 °C (4 °C/min), and detector temperature 300 °C.

### 4.4. Antioxidant Activity

The antioxidant activity of extracts was analyzed using the DPPH assay [44]. Different volumes of extracts were mixed with 95% and 90 μM DPPH solution. After the 60-min incubation at room temperature, absorption was measured at a wavelength of 515 nm. The antioxidant activity was expressed as IC_50_ value which represents the concentration of the extract which inhibits 50% DPPH radicals. All the measurements were performed in triplicate.

### 4.5. Animals and Treatments

Animal care and all experimental procedures were conducted in accordance with the Guide for the Care and Use of Laboratory Animal Resources edited by the Commission of Life Sciences, National Research Council. All procedures performed in the studies involving animals were in accordance with the ethical standards of the institution (University of Novi Sad; EK: II-2013-03; 01-160-5). For the purposes of this research, female mice of the Hannover National Medicinal Institute (Hann: NMRI) strain were used, aged 6–8 weeks, weighing 25 g ± 10%. Experimental animals were obtained from the laboratory of the Independent Department for Biochemistry, Laboratory Medicine Center, Clinical Center of Vojvodina (Novi Sad, Serbia). The animals were kept under strictly monitored conditions (temperature 25 °C, air humidity 30–50%, 12 h light/day cycles) in adequate cages and without limitations to access to food (LM2 with 19% protein, Veterinary Institute Subotica, Serbia) and water.

Extract SC was dissolved in olive oil in a concentration of 1% (SC1) and 5% (SC5). The SD was dissolved under sonication in water (1% (SD1) and 5% (SD5)), and filtered through A 0.45 mm membrane filter. The resulting solutions were kept refrigerated at a temperature of 4 °C. The extracts were administered intraperitoneally (i.p.).

The variability in the volume of administered doses was managed by adjusting the concentration to ensure a constant volume (2 mL/kg body weight).

During experimental work, mice were divided into groups of 6 mice through random selection and were treated in accordance with the following protocol:

- Group EAC—animals with implanted Ehrlich ascites carcinoma (EAC) cells treated with 2 mL/kg of saline, i.p. (n = 6).
- Group PRETREATMENT—animals pretreated with 2 mL/kg of the investigated extract, i.p. during seven days (n = 6).
- Group TREATMENT—animals were treated with 2 mL/kg of the investigated extract, i.p. seven days after the implantation of EAC (n = 6).
- Group POSTTREATMENT—animals post-treated with 2 mL/kg of the investigated extract, i.p. during seven days, seven days after implantation (n = 6).
- After 14 days from the day of implementation of EAC, all animals were sacrificed and the ascites was collected for further biochemical analyses.

### 4.6. Determination of Ascites Volume, Tumor Cell Number, and Cell Viability

The ascites from the abdomen was transferred to Krebs–Ringer phosphate buffer solution (0 °C, pH 7.4) and subjected to sequential centrifuging at 4500 rpm (MSE HIGH SPEED, 4 °C) and 12,000 rpm (Eppendorf 3200, 2.5 min) to obtain a dense cell suspension (1:1). The cell number was counted in a Neubauer chamber and expressed as number of cells/mm^3^. Cell viability was determined by the Trypan blue exclusion method and expressed as percentage of damaged cells.

### 4.7. Biochemical Assays

The samples were diluted with Krebs–Ringer phosphate buffer and the activities of antioxidant enzymes were determined in EAC cells by standard laboratory protocols. The activity of xanthine oxidase (XOD) was determined using the Bergmayer method [45], catalase (CAT) according to Beers and Sizer [46], peroxidase (Px) according to Simon et al. [47], glutathione peroxidase (GSHPx) according to Beutler et al. [48], and glutathione reductase (GR) according to Goldberg and Spooner [49]. The amount of reduced glutathione (GSH) was determined according to Beutler [50], as well as the intensity of lipid peroxidation (LPx) using the Buege and Aust protocol [51]. All the measurements were performed in triplicate.

### 4.8. Statistical Analysis

The activities are expressed as mean ± standard deviation. Mean values between the groups in the biochemical analyses were considered significantly different at the *p* < 0.05 confidence level, after performing a one-way single factor ANOVA, followed by Tukey and Bonferroni multiple comparison post hoc tests.

## 5. Conclusions

This study investigated the antiproliferative activity of supercritical and spray dried extracts of *S. montana* by using in vivo mice model. The impact of extracts on malignant cells was observed by applying the extracts as pretreatment, treatment, and posttreatment. It was determined that the time of application of extracts represents a significant parameter in the treatment of EAC cells. The assumption is that there was a significant development of tumors in the groups in which extracts were applied as posttreatment and that the application of extracts did not demonstrate to be an efficient approach. On the other hand, the extracts that were applied as pretreatment and treatment exhibited a far more significant effect on malign cells. The changes in the activities of biochemical parameters that represent markers of oxidative stress indicate that the malignant were exposed to oxidative stress when extracts were applied. Therefore, the results suggest that the extracts resulted in the generation of reactive oxygen species in malignant EAC cells.

The achieved results indicate the potential of *S. montana* in the prevention and treatment of malignant diseases. Furthermore, the results point to the necessity of the application of adequate environmentally-friendly procedures for the attainment of extracts that exhibit optimal effects and are safe at the same time. It is necessary to direct further investigation towards determining a precise mechanism of activity of extracts and their pharmacokinetic properties.

## Figures and Tables

**Table 1:** Impact of *S. montana* extracts on biochemical parameters in EAC cells.

Parameter	Group	EAC	SD1	SD5	SC1	SC5
XOD	Pretreatment	0.156 ± 0.009	0.142 ± 0.004 ^b^	0.151 ± 0.005 ^b^	0.807 ± 0.138 ^a,^*	0.822 ± 0.057 *^,a^
	Treatment		0.104 ± 0.011 ^c,^*	0.836 ± 0.069 ^b,^*	1.035 ± 0.064 ^a,^*	1.012 ± 0.014 ^a,^*
	Posttreatment		0.220 ± 0.014 ^a,^*	0.102 ± 0.005 ^b,^*	0.097 ± 0.012 ^b,^*	0.101 ± 0.004 ^b,^*
CAT	Pretreatment	0.503 ± 0.013	0.585 ± 0.019 ^a,^*	0.429 ± 0.002 ^b,^*	0.233 ± 0.016 ^c,^*	0.136 ± 0.002 ^d^*
	Treatment		0.636 ± 0.027 ^a,^*	0.138 ± 0.002 ^c,^*	0.194 ± 0.006 ^b,^*	0.191 ± 0.003 ^b,^*
	Post-treatment		0.316 ± 0.008 ^b,^*	0.698 ± 0.052 ^a,^*	0.713 ± 0.002 ^a,^*	0.742 ± 0.041 ^a,^*
Px	Pretreatment	0.325 ± 0.023	0.302 ± 0.016 ^c^	0.432 ± 0.010 ^c,^*	0.618 ± 0.003 ^b,^*	0.881 ± 0.019 ^a,^*
	Treatment		0.120 ± 0.006 ^c,^*	0.875 ± 0.035 ^a,^*	0.715 ± 0.004 ^b,^*	0.710 ± 0.002 ^b,^*
	Post-treatment		0.474 ± 0.035 ^a,^*	0.100 ± 0.008 ^b,^*	0.114 ± 0.023 ^b,^*	0.118 ± 0.007 ^b,^*
GR	Pretreatment	2.187 ± 0.107	2.115 ± 0.165 ^b^	3.012 ± 0.105 ^b,^*	4.957 ± 0.135 ^a,^*	5.707 ± 0.667 ^a,^*
	Treatment		1.202 ± 0.095 ^b,^*	5.627 ± 0.363 ^a,^*	5.172 ± 0.111 ^a,^*	5.195 ± 0.024 ^a,^*
	Post-treatment		3.930 ± 0.217 ^a,^*	1.195 ± 0.002 ^b,^*	1.254 ± 0.028 ^b,^*	1.284 ± 0.185 ^b,^*
GSHPx	Pretreatment	0.779 ± 0.048	0.796 ± 0.024 ^d^	0.933 ± 0.022 ^c,^*	1.302 ± 0.049 ^b,^*	1.697 ± 0.038 ^a,^*
	Treatment		0.474 ± 0.034 ^b,^*	1.91 ± 0.345 ^a,^*	2.043 ± 0.020 ^a,^*	2.007 ± 0.031 ^a,^*
	Post-treatment		1.131 ± 0.151 ^a,^*	0.395 ± 0.065 ^b,^*	0.401 ± 0.068 ^b,^*	0.421 ± 0.005 ^b,^*
GSH	Pretreatment	1.603 ± 0.110	1.417 ± 0.056 ^a,^*	1.358 ± 0.029 ^a,^*	0.871 ± 0.033 ^b,^*	0.884 ± 0.043 ^b,^*
	Treatment		1.513 ± 0.058 ^a,^*	0.926 ± 0.069 ^b,^*	0.621 ± 0.011 ^c,^*	0.635 ± 0.005 ^c,^*
	Post-treatment		1.105 ± 0.063 ^b,^*	1.801 ± 0.045 ^a,^*	1.799 ± 0.029 ^a,^*	1.842 ± 0.003 ^a,^*
LPx	Pretreatment	0.032 ± 0.008	0.030 ± 0.009 ^c^	0.041 ± 0.003 ^c^	0.100 ± 0.007 ^a,^*	0.080 ± 0.005 ^b,^*
	Treatment		0.022 ± 0.005 ^c^	0.042 ± 0.521 ^b^	0.077 ± 0.005 ^a^	0.083 ± 0.005 ^a^
	Post-treatment		0.056 ± 0.018 ^a,^*	0.011 ± 0.006 ^b,^*	0.019 ± 0.006 ^b,^*	0.014 ± 0.001 ^b,^*

Results are presented as a mean value ± SD from six mice. *—statistically significant difference compared to the EAC control group; different letters within a row indicate a significant difference between the samples in the same group at *p* < 0.05.

**Table 2:** GC/MS analysis of extract obtained by Sc-CO_2_ ((SC) extract) and essential oil isolated from spray-dried (SD) extract (relative percentage; %) [32,33].

Compound	SC	SD
α-Terpinene	0.15	n.i.
*p*-Cymene	3.96	0.36
γ-Terpinene	0.80	n.i.
α-Terpineol	n.i.	0.21
Eucalyptol	0.42	n.i.
*Trans*-sabinene hydrate	0.33	n.i.
*Cis*-sabinene hydrate	0.15	n.i.
Linalool	0.29	0.18
Borneol	1.56	n.i.
Terpinen 4-ol	0.84	0.42
Carvacrol	78.61	71.82
*Trans*-caryophyllene	2.40	0.24
Caryophyllene oxide	1.26	1.31
α-Amorphen	0.46	n.i.
β-Bisabolene	0.74	n.i.
γ-Cadinene	0.53	n.i.
δ-Cadinene	0.78	n.i.
β-Cadinene	n.i.	0.24
Spatulenol	n.i.	0.21
Heptakosane	0.17	n.i.
Nonakosane	0.19	n.i.

n.i. not identified.

**Table 3:** Biochemical parameters of control group and the groups treated with the SD1 and SC1 extracts [36].

Group	XOD	CAT	Px	GR	GSH-Px	GSH	LPx
Control	1.93 ± 0.02 ^a^	9.56 ± 0.37 ^b^	10.68 ± 1.38 ^b^	6.13 ± 0.07 ^a^	8.70 ± 0.38 ^a^	4.98 ± 0.16 ^a^	3.04 ± 0.07 ^a^
SC	1.87 ± 0.03 ^ab^	13.4 ± 0.59 ^a^	14.88 ± 0.38 ^a^	7.03 ± 0.46 ^a^	7.54 ± 2.23 ^a^	4.99 ± 0.17 ^a^	2.62 ± 0.54 ^a^
SD	1.86 ± 0.03 ^b^	12.37 ± 0.18 ^a^	14.57 ± 0.80 ^a^	6.53 ± 1.61 ^a^	6.67 ± 1.06 ^a^	4.92 ± 0.39 ^a^	2.21 ± 0.24 ^a^

Values are expressed as mean ± standard deviation for six mice. Activities of xanthine oxidase (XOD), catalase (CAT), peroxidase (Px), glutathione reductase (GR) and glutathione peroxidase (GSHPx) are expressed in nmol/mg of protein min-1. Content of hepatic reduced glutathione (GSH) is expressed in nmolGSH/mg of protein. Intensity of lipid peroxidation (LPx) is expressed in nmol/MDA/mg of protein; MDA, malonyldialdehyde. Different letters within a column indicate a significant difference between the samples at *p* < 0.05.

## Appendix A

**Table A1:** Impact of *S. montana* extracts on the volume of ascites.

Group	EAC Ascites Volume (mL)			
EAC Control	7.117 ± 0.458			
Extract	SD1	SD5	SC1	SC5
Pretreatment	2.267 ± 1.178 ^a,^*	3.283 ± 1.301 ^a,^*	2.267 ± 0.674 ^a,^*	1.000 ± 0.469 ^a,^*
Treatment	2.167 ± 0.288 ^ab,^*	0.817 ± 0.147 ^b,^*	2.933 ± 1.147 ^a,^*	2.267 ± 1.065 ^ab,^*
Post-treatment	3.083 ± 0.382 ^b,^*	9.657 ± 0.359 ^a,^*	9.851 ± 0.932 ^a,^*	9.622 ± 0.954 ^a,^*

Results are presented as a mean value ± SD from six mice. *—statistically significant difference compared to the EAC control group; different letters within a row indicate a significant difference between the samples in the same group at *p* < 0.05.

**Table A2:** Impact of *S. montana* extracts on the viability of EAC cells.

Group	EAC Cells Viability (%)			
EAC Control	7.243 ± 1.425			
Extract	SD1	SD5	SC1	SC5
Pretreatment	7.183 ± 1.705 ^a^	6.267 ± 0.965 ^a^	5.967 ± 0.339 ^a^	6.250 ± 1.205 ^a^
Treatment	4.817 ± 1.042 ^a,^*	6.550 ± 0.509 ^a^	6.733 ± 0.857 ^a^	6.200 ± 1.131 ^a^
Post-treatment	6.533 ± 0.476 ^a^	7.650 ± 0.874 ^a^	7.499 ± 0.356 ^a^	7.586 ± 1.023 ^a^

Results are presented as a mean value ± SD from six mice. *—statistically significant difference compared to the EAC control group; different letters within a row indicate a significant difference between the samples in the same group at *p* < 0.05.

**Table A3:** Impact of *S. montana* extracts on the number of EAC cells.

Group	EAC Cells Number/mm^3^			
EAC Control	119 583.333 ± 6 755.862			
Extract	SD1	SD5	SC1	SC5
Pretreatment	196,583.333 ± 17,133.058 ^a,^*	158,833.333 ± 9739.952 ^ab,^*	147,000.000 ± 22,858.259 ^b^	125,583.333 ± 21,731.122 ^b^
Treatment	247,000.000 ± 44,340.730 ^a,^*	114,333.300 ± 1505.545 ^b^	112,166.700 ± 16,898.720 ^b^	108,000.000 ± 17,378.150 ^b^
Post-treatment	141,833.333 ± 24,717.740 ^b,^*	346,569.58 ± 34,156.81 ^a,^*	374,511.22 ± 11,568.13 ^a,^*	361,581.47 ± 8455.36 ^a,^*

Results are presented as a mean value ± SD from six mice. *—statistically significant difference compared to the EAC control group; different letters within a row indicate a significant difference between the samples in the same group at *p* < 0.05.
